# P-738. Doxycycline for Syphilis Treatment at A VA Medical Center During a Benzathine Penicillin Shortage

**DOI:** 10.1093/ofid/ofaf695.949

**Published:** 2026-01-11

**Authors:** Richard L Oehler, John F Toney, Tiffany Ward, Sreenath Varma

**Affiliations:** James A Haley Veterans Hospital, Tampa, FL; James A Haley Veterans Medical Center, Tampa, Florida; James A. Haley Veterans' Hospital, Tampa, Florida; University of South Florida, Tampa, Florida

## Abstract

**Background:**

U.S. Syphilis cases are rising, including in Florida. Benzathine Penicillin (BP) is the preferred treatment of syphilis with a clinical success rate of 90-100%. Doxycycline (Doxy) remains an alternative treatment option, with a 83-100% rate of clinical success. The Tampa VA Medical Center faced a national BP shortage beginning in April 2023 due to manufacturer delays and rising treatment need. Although the FDA temporarily allowed importation and use of benzathine benzylpenicillin (Extencilline™), federal health care facilities continued faced procurement restrictions, and BP was rationed. Oral doxy 100 mg orally twice daily became our preferred syphilis treatment for 14 months.

**Figure 1:**
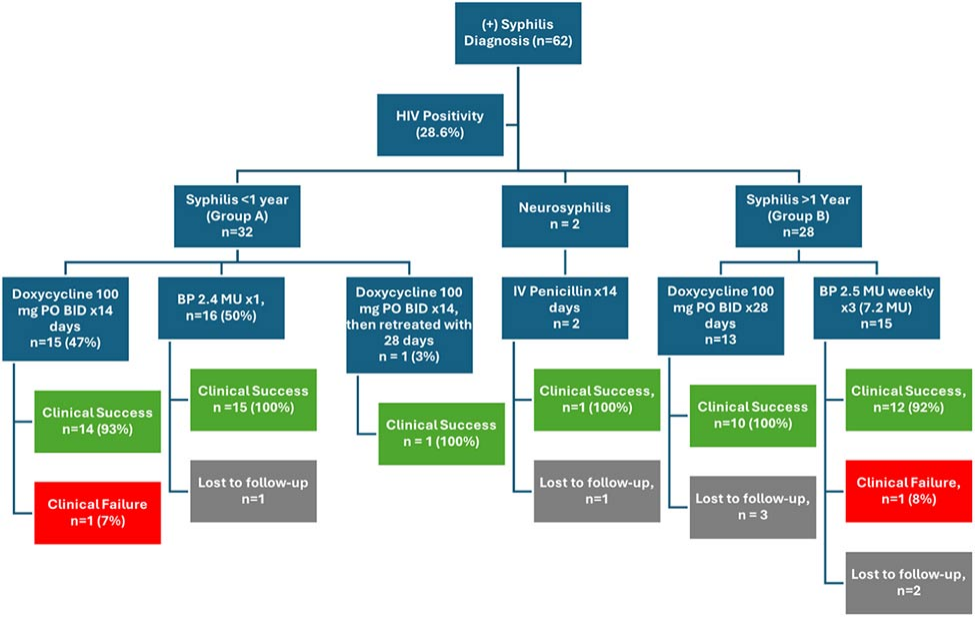
Patient Clinical Response based on Syphilis Treatment

**Figure 2:**
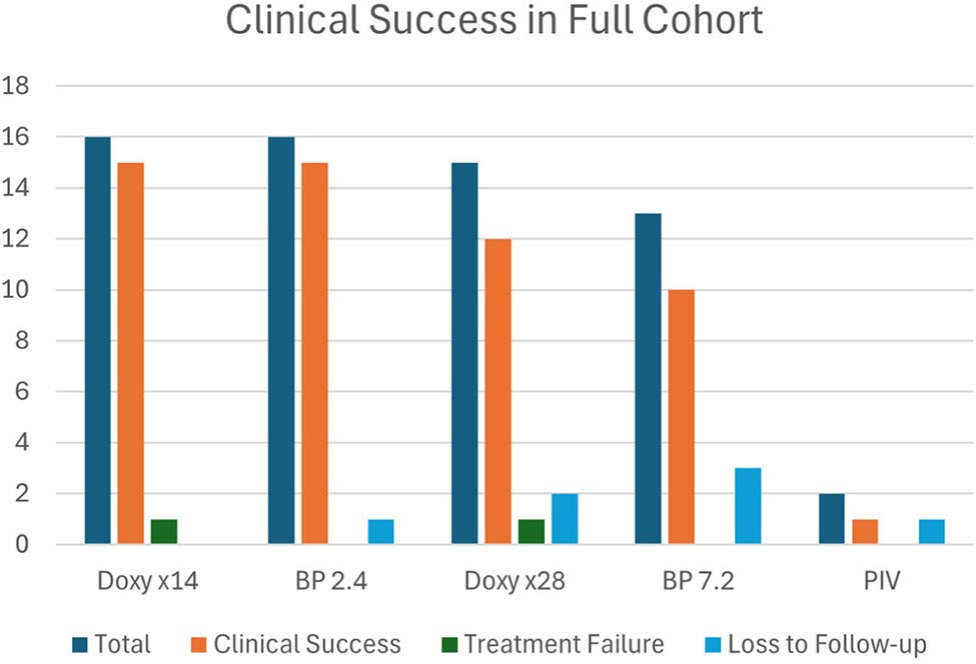
Clinical Success in the Full Cohort

**Methods:**

We retrospectively reviewed all veterans treated for syphilis at our center between 1/1/23 and 12/31/24. Cases identified had a positive nontreponemal test and an elevated rapid plasma reagin (RPR) titer of ≥ 1:1. If RPR positive, a > 4x rise in the RPR titer met criteria for retreatment. Sixty-two patients (pts) met criteria. Data collected included date/value of RPR titer, syphilis stage, regimen used, HIV status, and treatment response as defined as a > 4x reduction in titer.

**Figure 3:**
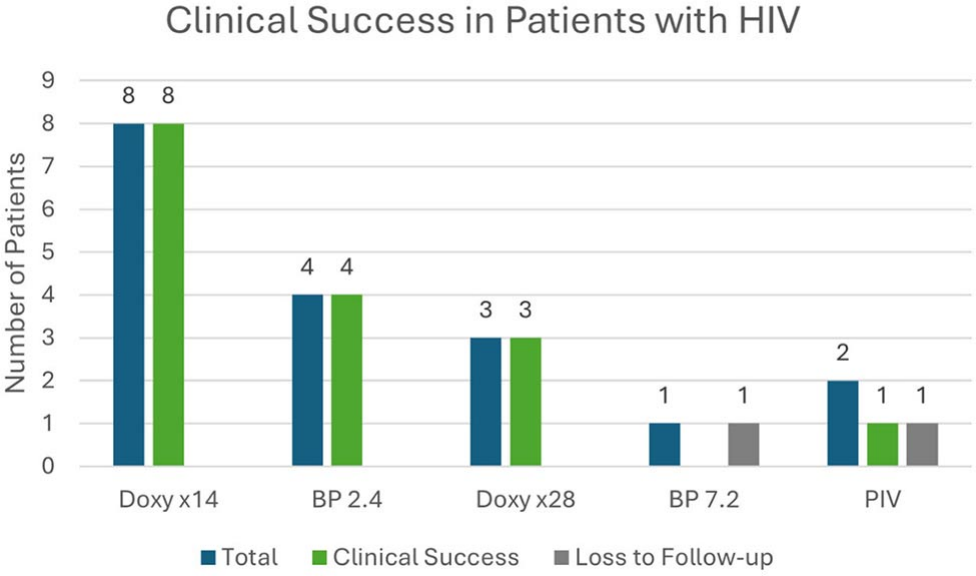
Clinical Success in Patients with HIV

**Results:**

Of 62 pts, there were 32 pts w/syphilis < 1 yr, and 28 pts w/syphilis > 1yr, and 2 pts with neurosyphilis (NS) (Fig. 1). The Doxy x 14 days success rate was 93% and the BP 2.4 million units (MU) success rate was 100%. One patient received Doxy x 14 days and was retreated x 28 days due to noncompliance. The success rate for Doxy x 28 days was 92% and for BP 7.2 MU was 100%. One patient in the BP 2.4 MU group, 2 in the BP 7.2 MU group, 3 in the Doxy x 28-day group, and 1 NS patient did not follow up (Fig. 1). HIV positivity was 28.6%. Overall, clinical success rates exceeded 90% for Doxy x 14 days and Doxy x 28 days in comparison to BP 2.4 MU and BP 7.2 MU, respectively (Fig. 2). The clinical success rate in the HIV subpopulation for the Doxy x14, Doxy x28, and BP 2.4 MU group was 100%. One HIV patient was lost to follow-up in the BP 7.2 MU group and in the NS group (Fig. 3).

**Conclusion:**

In veterans with syphilis, responses were similar in both the Doxy and BP groups during the review period, with both achieving greater than 90% clinical success, including in an HIV+ cohort. In the face of Penicillin supply chain irregularities, Doxy remains a highly effective alternative to BP.

**Disclosures:**

All Authors: No reported disclosures

